# Challenges and opportunities for real-world evidence in clinical oncology—a view from the UK: proceedings of a national workshop

**DOI:** 10.1016/j.esmorw.2024.100089

**Published:** 2024-11-18

**Authors:** M. Craddock, C. Dempsey, D. Abdulwahid, J.P.C. Baldwin, K. Banfill, A. Carver, A. Chaturvedi, S. Cheeseman, G.W. Cowell, M. Daly, A. Dekker, S.R. Dubash, S. Duffield, I. Fornacon-Wood, M.A.C. Garcia, P. Goodley, H. Green, R.J. Holley, S. Ingram, S. Jones, J. Kennedy, A. Lighterness, C.K. McGarry, O. McLaughlin, R. Mir, B.W. Papiez, D.E.J. Snelling, S. Theophanous, S. Warren, K. Zucker, G. Price, C. Faivre-Finn

**Affiliations:** 1 Radiotherapy Related Research Group, Division of Cancer Sciences, School of Medical Sciences, University of Manchester, Manchester, UK; 2 The Christie NHS Foundation Trust, Manchester, UK; 3 Leeds Teaching Hospitals Trust, Leeds, UK; 4 University Hospitals Birmingham NHS Foundation Trust, Birmingham, UK; 5 Queen Elizabeth University Hospital, Glasgow and School of Cancer Sciences, University of Glasgow, Glasgow, UK; 6 Department of Radiation Oncology (MAASTRO), GROW School for Oncology and Reproduction, Maastricht University Medical Centre, Maastricht, The Netherlands; 7 Mount Vernon Cancer Centre, London, UK; 8 National Institute for Health and Care Excellence (NICE), London, UK; 9 Edinburgh Cancer Centre, Edinburgh, UK; 10 Division of Immunology, Immunity to Infection and Respiratory Medicine, University of Manchester, Manchester, UK; 11 Royal Marsden NHS Foundation Trust, London, UK; 12 Institute of Cancer Research, London, UK; 13 Proton Clinical Outcomes Unit, Proton Beam Therapy Centre, The Christie NHS Foundation Trust, Manchester, UK; 14 Radiotherapy Physics, Northern Ireland Cancer Centre, Belfast Health and Social Care Trust, Belfast, UK; 15 Patrick G. Johnston Centre for Cancer Research, Queen’s University Belfast, Belfast, UK; 16 Big Data Institute, University of Oxford, Oxford, UK; 17 Northern Centre for Cancer Care Freeman Hospital, Newcastle upon Tyne, UK; 18 University of Leeds, Leeds, UK

**Keywords:** real-world data, patient data, evidence-based radiotherapy, clinical trials, data science

## Abstract

Real-world data (RWD) are defined as information collected about patients as a routine part of treatment. To understand the status of RWD initiatives in oncology in the UK, an online survey and in-person workshop were conducted which aimed to characterise current perceptions of RWD, establish where real-world evidence (RWE) could support unmet clinical need, and to identify the barriers and solutions to obtaining this evidence. Self-selecting health care professionals including oncologists, physicists, radiographers, and health data researchers, as well as patient representatives, participated in an anonymous survey (*N* = 55) and/or a 1-day workshop (*N* = 46). The workshop consisted of introductory presentations followed by three 1 hour grouped breakout sessions. An inductive thematic analysis synthesizing the outcomes of the survey and workshop was carried out *post hoc*. Despite issues of perceived poor data quality and the prevalence of unstructured data, 92% of survey respondents recognised the potential of RWD to provide novel evidence. Suggested applications of RWE were validation of trial results in the general population, continuous evaluation of new technologies, decision-making in rare disease groups, and resource allocation. Barriers to progression of RWD initiatives identified were data accessibility, data quality, and prioritisation. Potential solutions include streamlining information governance processes, training staff in data science skills, and demonstrating clinical benefit. The potential of RWD to provide novel evidence is strongly recognised in the UK radiotherapy community. While barriers to progress were identified, none of them are insurmountable. To move forwards, the profile of RWE needs to be elevated to attract higher prioritisation and resourcing.

## Highlights

• The UK radiotherapy community strongly recognises the potential of real-world data.
• Barriers to wider usage include data accessibility, data quality, and prioritisation.
• Information governance processes need to be streamlined to improve data access.
• Training staff in data science skills would contribute to improving data quality.
• Higher prioritisation is needed to realise the potential of real-world data.

## Introduction

Real-world evidence (RWE) has been proposed as a potentially valuable source of evidence to support clinical decision-making in oncology.^1^^,^^2^ RWE is derived from the analysis of curated real-world data (RWD) which, while not subject to the rigorous quality control measures typical of trial datasets, offer the inherent advantages of already being captured as part of routine clinical care and also being inclusive of all patients treated. Recognising the potential of RWD, several national and international initiatives have been established to generate comprehensive repositories of RWD. These include Data-Can, the European Network of Cancer Registries and Consore.^3^, ^4^, ^5^ Despite this, RWE has yet to demonstrate significant impact on clinical practice in radiotherapy and phase III randomised controlled trials remain the gold standard of clinical evidence.^6^

There is ongoing debate around the breadth of what may be considered as RWD, but a working definition adopted in this paper may be expressed as: patient data collected as a routine part of standard clinical care.^6^^,^^7^ The US Food and Drug Administration (FDA) and the National Institute for Health and Care Excellence (NICE) both employ the broadest definition of RWD as data gathered ‘outside the context of a highly controlled clinical trial’.^8^^,^^9^ This includes data gathered prospectively to address specific research questions and may even include interventional studies, such as pragmatic clinical trials. Ultimately, the debate over the definition of RWD centres on whether a data mining or data farming approach is adopted. The former involves the analysis of pre-existing health care data sources, while the latter describes the strategic development of reliable databases designed to support research and its translation to clinical practice.^10^

Over the last 20 years, radiotherapy has undergone significant technological evolution with the implementation of both intensity modulation and image guidance as standard of care leading to more precise and effective treatments with fewer side-effects.^11^^,^^12^ Ensuring the delivery of optimal radiotherapy treatments with this rapid pace of progress requires the generation of evidence on a shorter timescale and at a lower cost than can be supported by conventional clinical trials. Using real-world outcomes data to drive continuous improvement cycles in the rapid learning process may be able to meet this need and avoid the adoption of changes in practice without ongoing formal clinical evaluation.^13^

While RWE is often presented as an alternative to clinical trials, in reality it is a complementary approach to evidence generation.^1^ Fewer than 1 in 20 adult cancer patients participate in clinical trials due to a complex mix of structural, clinical, and attitudinal factors which differ across ethnic and socioeconomic groups.^14^, ^15^, ^16^, ^17^ This, combined with the common use of restrictive eligibility criteria which tend to favour younger and fitter patients, can result in narrow study populations which lack the heterogeneity of the whole clinical cohort.^18^^,^^19^ This results in high internal validity of trial results, but questionable generalisability of the findings to the general patient population.^20^ Here, RWD can play a valuable role in determining treatment effectiveness in clinically realistic patient groups.^21^^,^^22^

There are also many changes in practice for which clinical trials are not suitable. A key example is the introduction of a new technique which is considered superior to the current standard of care and therefore cannot be considered to provide clinical equipoise. Additionally, any change in practice which evolves over time, due to an associated learning curve, challenges conventional clinical trial methodology.^23^^,^^24^ As such, over-reliance on trials can lead to the adoption of changes in practice without an appropriate evidence base or on-going evaluation. Analysis of RWD may be able to provide the evidence to bridge this gap.

This study aims to establish the current status of RWD research and the impact of RWE on clinical practice in the UK, identify the opportunities for RWE to support unmet clinical need, as well as the barriers and solutions to achieving this. We report the outcomes of a survey and an in-person national workshop attended by oncologists, clinical scientists, therapeutic radiographers, digital health services staff, and patient representatives.

## Methods and materials

### Survey

To gain a basic overview of how the UK oncology community views RWE and inform the design of the in-person workshop, a short online survey was conducted. Self-selecting participants were invited to respond anonymously to the survey subject to their involvement in the collection, use, or management of RWD in the field of clinical oncology. These self-selection criteria were presented on the pre-survey landing page and included in all communications related to the survey. The survey had a total of 20 questions, with a subset pertaining to clinical scenarios restricted to clinicians. The survey was designed by the workshop organisation team and tested before release both by internal and external staff members on an *ad hoc* basis. The survey was disseminated via the medical physics mailbase,^25^ an NHS head of clinical oncology services mailing list, the Cancer Research UK (CRUK) RadNet AI and computational modelling working group, HDR-UK/Datacan, and various internal mailing lists at the Christie NHS Foundation Trust. The full question list is provided in the Supplementary Materials, available at https://doi.org/10.1016/j.esmorw.2024.100089.

The Qualtrics platform was used to deliver the survey and record responses.^26^ Results of the survey were presented in the first session of the in-person workshop.

### Workshop

The full-day in-person workshop consisted of three sessions focussing on: (i) perceptions of the use of RWD, (ii) clinical questions that could be addressed with RWE, and (iii) the datasets needed to address these questions.

Each session began with opening presentations, designed to provide balanced information and context to inform the subsequent 1-h breakout sessions. During the breakout sessions, five groups of around nine delegates discussed the session topic and reported their conclusions to the entire cohort. A complete list of the presentations delivered is provided in the Supplementary Materials, available at https://doi.org/10.1016/j.esmorw.2024.100089. A nominated chair was asked to keep discussions on-topic and ensure everyone’s voice was heard, with discussion points and themes captured using flipcharts by a nominated scribe for the session.

Workshop participants were self-selecting according to the same criteria as employed for the survey which was presented on the Eventbrite registration page and included in all communications related to the workshop. Discussion groups were pre-allocated to balance the representation of staff groups as far as possible and were fixed for the duration of the workshop. A summary of key themes identified during the course of the day was presented at the close of the workshop, with time for further questions and discussion. A full running order for the day is provided in the Supplementary Materials, available at https://doi.org/10.1016/j.esmorw.2024.100089.

The workshop was listed on EventBrite^27^ and advertised through the same channels as used for the survey, with additional promotion via National Cancer Research Institute group leads, the Pankhurst Institute, the Digital Futures network at the University of Manchester, and in person at the Rapid-RT stakeholder event and at the CRUK Data meeting held on the 4 and 14 July 2023, respectively.

### Analysis

Survey responses were almost exclusively captured in a closed, structured format and each question was simply presented graphically in the most appropriate format. Clinicians were asked to identify a clinical scenario that is poorly supported by current evidence (if any). Their responses were captured as free text, which was then manually coded by disease site and clinical query.

To minimise the potential for any preconceptions of the authorship to influence the reporting of the outcomes of the workshop, an inductive thematic analysis approach was adopted. As described by Braun and Clarke,^28^ the inductive approach involves deriving themes and meaning purely from the data, devoid of any pre-defined coding or personal preconceptions. As such, the only pre-organisation of the outputs from the workshop, which consisted of flip chart notes and a summary of key discussion points presented on the day, was to separate them into the workshop sessions they originated from.

The analysis process consisted of firstly digitising the flip chart notes, followed by categorising each of the entries into major themes (such as data quality) and their sub-themes (such as lack of data quality control). Themes were then ranked by the number of times they occurred across discussion groups, with higher ranked themes and sub-themes preferentially selected for inclusion in the results and discussion.

All attendees of the workshop were given the opportunity to provide written feedback on a pre-submission version of the article and indicate whether they wished to be included in the author list. Attendees who failed to respond were omitted from the authorship.

## Results and discussion

### Survey response and workshop attendance

A total of 74 survey responses were received in the period of 27 March 2023 to 29 August 2023. A total of 19 respondents provided only their job title and these responses were excluded from the analysis. Of the 55 remaining responses, 51 were complete, with four respondents only completing the first group of questions pertaining to existing attitudes to, and knowledge of, the collection and use of RWD. Respondents represented clinical scientists (31%), oncologists (29%), therapeutic radiographers (12%), digital health services (4%), data entry (2%), and other staff groups (22%), including health technology assessment, patient advocates, and pharmacists.

**Figure 1:**
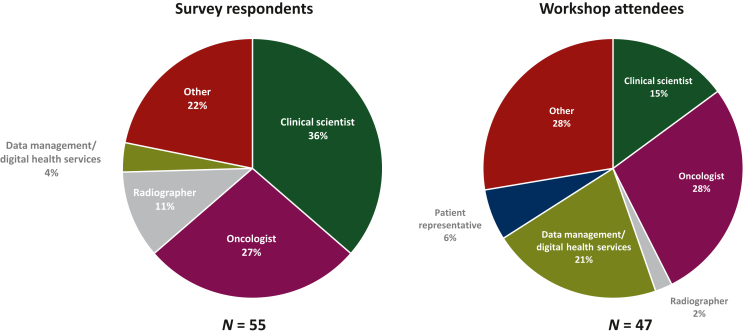
Breakdown of survey respondents and workshop attendees by staff group.

The workshop was held on the 6 September 2023 in Manchester and was attended by 47 people consisting of oncologists, physicists, radiographers, patient representatives, data scientists, and health data researchers representing 17 institutions from across the UK. Figure 1 shows the distribution of staff groups represented at the workshop.

The sections that follow report the outcomes of the workshop discussions organised by the three major sessions of the day. Due to the similarity of the themes addressed and for readability, the results of the survey and workshop themes are presented collectively.

### Session 1: perceptions of the use of RWD

#### Quality

**Figure 2:**
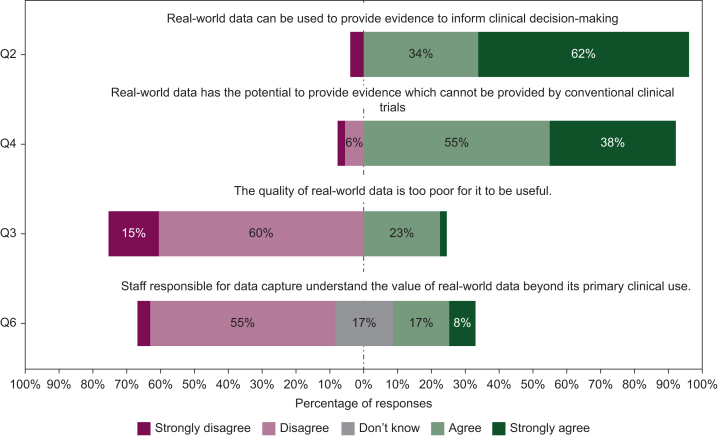
**Perceptions of real-world data in the UK oncology community.** Please note only Q6 included ‘Don’t know’ as a response option.

**Figure 3:**
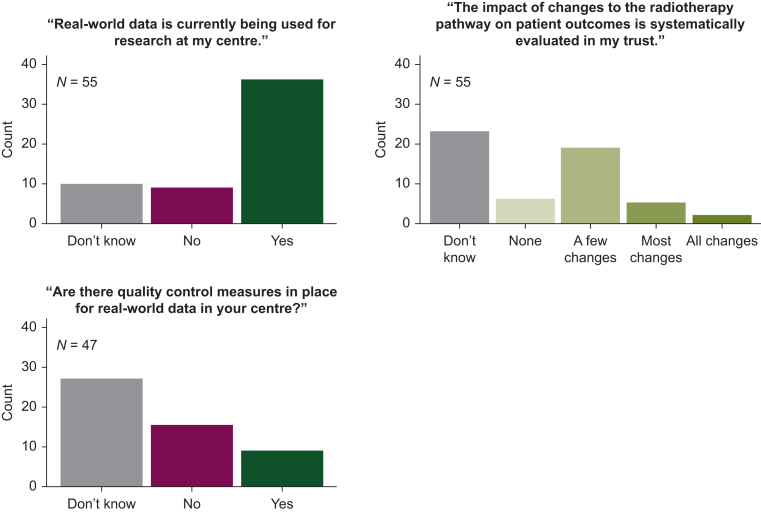
Current usage and quality control of real-world datasets in the UK.

As shown in Figure 2, the potential of RWD to provide both useful evidence to inform clinical decision-making and novel evidence, beyond that which can be derived from conventional clinical trials, was strongly recognised by survey respondents, with >90% agreeing or strongly agreeing with both statements. A less favourable view of RWD quality was expressed, with 25% of respondents agreeing or strongly agreeing that the quality of RWD is too poor to be useful, and this was reflected in the group discussions. Data quality issues such as incompleteness, erroneous data, and loss of data on transfer were highlighted by all groups. In addition, a lack of awareness of how to appropriately quality assure RWD emerged as a theme. This was reinforced by the majority of survey respondents (53%) being unaware of whether RWD quality control measures were in place at their centre (Figure 3). Within the discussion groups, concerns regarding the resource implications associated with quality assurance were expressed. Attendees from institutions working with organisations who provide data curation services, such as DATA-CAN and Flatiron Health, suggested that this model could be adopted more broadly to facilitate the appropriate management and curation of RWD without placing additional demands on clinical departments.^3^^,^^29^

#### Engagement

The engagement of staff responsible for the collection of RWD was recognised as an important human factor which drives data quality. Some 61% of survey respondents did not consider that staff understood the value of RWD and group discussions on this issue focused on potential solutions. The streamlining of data collection workflows to minimise the impact on workload was commonly suggested and given that electronic record keeping is a known risk factor for burnout in oncologists, has potential to be an effective strategy for improving engagement.^30^ Demonstration of the clinical impact of RWE was also suggested as a method of improving staff engagement, with the cardiac toxicity studies conducted at the Christie and the Cancer Outcomes and Services Dataset (COSD) suggested as potential examples.^13^^,^^31^

#### Structure

Three of five groups raised issues relating to the lack of structure of RWD. While there was significant diversity in the systems and processes used to capture data, there was a clear consensus that large amounts of oncology data are currently stored in an unstructured format, with some centres still reliant on paper records. Unstructured data refers to data without a pre-defined format, which cannot be directly queried and therefore are far more difficult to use for any secondary purpose.

The three most common forms of unstructured data reported by the survey were free text in electronic health records, letters or reports stored as PDFs, and scans of physical documents. Several instances of data which were structured at source being stored in an unstructured format were discussed, typically following export as a PDF. This highlights the need for standardised data export and import mechanisms for all oncology systems to facilitate the transfer of structured data, although this may also speak to a need for training in the importance of persisting structured datasets. Use of artificial intelligence-based natural language processing algorithms to extract structured data was also discussed, with some centres already using or evaluating commercial solutions.

**Figure 4:**
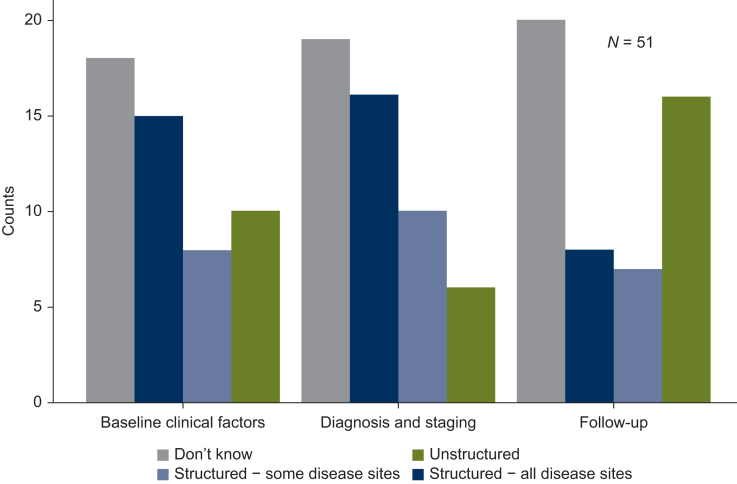
Data storage structure by category of baseline clinical factors, diagnosis and staging, and follow-up.

As shown in Figure 4, the survey probed how RWD is stored across the categories of: baseline clinical factors, diagnosis and staging, and follow-up. While ‘don’t know’ was the most common response across all categories, the majority of other respondents stated that baseline clinical factors and diagnosis and staging data for some or all disease sites were stored in a structured format (23 versus 10 and 26 versus 6 responses for structured versus unstructured data in baseline clinical factors and diagnosis and staging, respectively). This trend notably reversed for follow-up data, suggesting that unstructured data may be more prevalent in datasets captured after treatment, which typically hold the outcomes data used in RWE studies. As such, it is important that efforts to improve data collection methods address the follow-up phase, as well as pretreatment and on-treatment data.

#### Accessibility/governance

**Figure 5:**
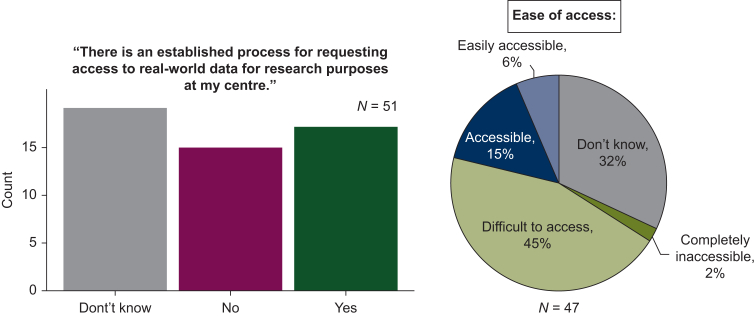
Accessibility of real-world datasets in UK oncology centres.

Poor accessibility of RWD was the strongest theme to emerge from both the first breakout session and the survey. Information governance processes, lack of support services, lack of interoperability between systems, and difficulty finding specific records were equally cited as the causes of 47% of respondents categorising access either as difficult or impossible (Figure 5). Group discussions highlighted inefficient and unclear governance processes as the primary cause of poor data accessibility. Sharing best practice examples of governance frameworks from centres was deemed to have solved these problems and was suggested as a potential solution. Given the difficulty of accessing RWD from within the host organisation, it was generally acknowledged that gaining external access to RWD is even more challenging, although federated learning approaches could circumvent this issue.^32^

### Session 2: clinical questions that could be addressed by RWE

Three out of five groups began the second breakout session with discussion of the shortcomings of conventional clinical trials and used this as a basis to identify potential areas for RWE studies. The cost, long-time span, and non-representative patient cohorts were the most commonly recognised limitations. Validation of clinical trial results in the general patient population was considered to be a valuable application of RWD, potentially fulfilling the role of costly phase IV clinical trials which are commonly conducted for new cancer drugs, but rarely for changes in radiotherapy practice.^33^^,^^34^ Studies within patient groups typically underrepresented in trials such as children, the elderly, the co-morbid, and ethnic minorities were also proposed.

Adoption and optimal usage of novel technologies such as adaptive radiotherapy and magnetic resonance-guided radiotherapy platforms were also commonly discussed as an area where RWE could play a key role. Here, the rapid evolution of technology over time poses a major challenge to evaluation through conventional trials. In contrast, RWD offer the potential to apply novel methodologies, such as rapid learning, in which an intervention is made and iteratively optimised by using real-world outcomes data to drive quality improvement cycles.^35^ More generally, the R-IDEAL framework, an adaptation of the IDEAL framework for the evaluation of surgical innovation, provides a structure to support the coordinated, evidence-based introduction of new technologies. It has been adopted by the MR-Linac consortium.^36^

Patient representatives raised the impact of long-term toxicity on the quality of life of cancer survivors, an area which is currently poorly researched and of increasing importance as cancer survival rates improve.^37^ While the timespan between treatment and long-term toxicity makes conventional trials impractical, RWE studies, collecting patient-reported outcome measures (PROMs) could play a role in firstly reporting long-term toxicity and ultimately guiding treatment optimisation to minimise long-term toxicity. Stage 4 of the R-IDEAL framework pertains to long-term evaluation and calls for the establishment of prospective registries for innovations. RWD may be able to fulfil this role whilst having the advantage of not being limited to specific innovations.

Clinical scenarios which could be supported by RWE suggested by clinicians included analysis of outcomes in recurrent disease, as well as patient groups commonly underrepresented in trials; evaluation of late effects; and the use of electronic PROMs to assess skin reaction in breast cancer. Treatment of rare diseases, particularly in skin cancer, was raised as an area where the larger cohorts of real-world datasets may be able to provide the missing evidence required to guide clinical decision-making. Optimal treatment of patients with chronic conditions, such as cachexia and myasthenia gravis, were also suggested as a clinical query which RWE could support. Some 82% of the respondents who submitted a clinical scenario felt that RWD could provide useful evidence to support their specific scenario.

### Session 3: datasets required

The variability in current infrastructure for RWD capture discussed in the first break-out session and in particular, the continued reliance of some centres on paper records, was recognised as a barrier to the development of high-quality national real-world datasets. A national audit was proposed as a potential solution to better characterise this problem, but additional resource, personnel, and training will be required to harmonise the data collection and storage infrastructure of all centres.

**Figure 6:**
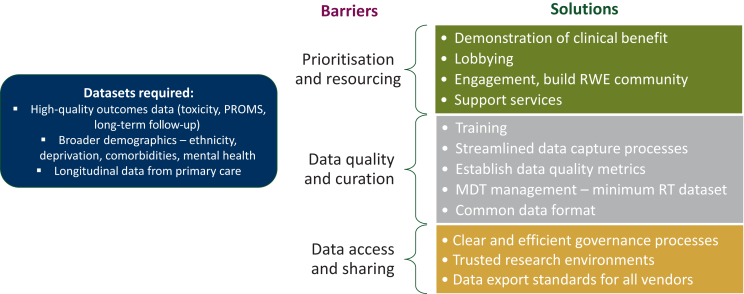
**Summary of the major themes to emerge from the workshop group discussions.** MDT, multidisciplinary team; PROMS, patient-reported outcome measures; RT, radiotherapy; RWE, real-world evidence.

A lack of prioritisation and resourcing was recognised as a common cause of many of the previously discussed shortcomings of current RWD. While research based on RWD is generally considered to be less costly than conventional clinical trials, discussion groups recognised that capturing and curating high-quality datasets requires infrastructure, expertise, and time, all of which have associated costs. Provision of data science training for staff, funding of support services, and infrastructure changes such as implementing trusted research environments (TREs) were all recognised as costs associated with the development of RWD for research, as summarised in Figure 6.

For RWD to be given a higher priority in the UK, many groups expressed that evidence of clinical benefit was a prerequisite. Lobbying of key decision-makers was also discussed as a strategy to elevate the profile of RWE, although there was debate on whom the appropriate person or body to lobby would be, with NHS Digital, NHS England, and the government all suggested. It was also felt that developing an RWE-focussed community within oncology would help to create broader awareness.

Several groups raised the need for the establishment of both a common data format for oncology, which would ease joining of multicentre datasets, and a minimum radiotherapy dataset to be held by all UK centres. It should be noted, however, that multiple national cancer datasets compiled from mandatory reporting by all NHS England trusts have already been established by the National Disease Registration Service and are being used in a wide range of research studies.^38^, ^39^, ^40^, ^41^, ^42^ These include the national RadioTherapy DataSet (RTDS), which contains patient, tumour, and radiotherapy treatment level information; the COSD, which also includes non-radiation treatment data, pathology and outcomes; as well as the National Cancer Registration and Analysis Service (NCRAS), which aggregates multiple datasets to support policy-making and research.^43^, ^44^, ^45^ As such, this theme may highlight a lack of awareness, or equally a lack of publicization of existing national datasets, which may be under-utilised as a result. It should be noted, however, that the existing UK national oncology databases suffer from the major limitation of not holding any imaging or radiotherapy plan data, which are prerequisites for data mining and radiomics studies.

Concerns regarding the ethical implications of developing national datasets were also raised. It was noted that gathering ‘nice-to-have’ data for potential future research raises ethical dilemmas due to the lack of a clearly defined intended usage, thereby challenging conventional models of consent.^46^ The national data opt-out is currently the main consent process for RWD used in the UK. The Royal College of Radiologists (RCR) consent forms, however, which include an opt-in for data use for educational, audit, and research purposes are widely used in the UK.^47^^,^^48^ Whether these approaches will be deemed sufficient when RWE begins to significantly impact on clinical decision-making and patient care is a matter of debate. The potential for re-identification of individuals from broad anonymised datasets also poses a major risk to patient privacy, particularly with genetic data.^49^

#### Strengths and limitations

This study presents for the first time, the perceptions of a broad range of stake holders in the field of clinical oncology in the UK on the use of RWD, the clinical questions RWE could support, as well as the barriers and solutions to obtaining this evidence. While extensive literature exists on RWD in oncology, to date most studies have focused on methodology, practical examples of RWE generation, and high-level reviews of the state of the field. An exception to this is the international survey of clinicians conducted by the European Organisation for Research and Treatment of Cancer (EORTC) which reported similarly positive findings regarding perceptions of RWE, but also highlighted that nearly 50% of respondents felt that RWE could not fully address evidence gaps created by new anticancer therapies, with 77% still viewing RCTs as the gold standard of clinical evidence.^50^

The survey respondents and workshop attendees were self-selected, which may have introduced a source of bias. It is reasonable to expect that individuals with a pre-existing interest in RWD were more likely to participate, potentially leading to over-representation of such individuals. It should be noted, however, that a pre-existing interest does not necessarily imply a more positive or negative view of RWD initiatives. Furthermore, the diverse representation of staff groups from 17 different institutions helps to mitigate the impact of any potential cohort associated with self-selection.

### Conclusion

The UK oncology community recognises the potential of RWD to provide useful evidence to support clinical decision-making. RWD are already being used for research purposes in many centres, but to make further progress the major barriers of data quality, structure, standardisation, and accessibility must be addressed. Higher prioritisation, engendered through community engagement, lobbying, and demonstration of clinical benefit, as well as additional resourcing to fund staff training, support services and infrastructure changes, is needed to realise the full potential offered by RWE.
