# Supplementary material for: Challenges and opportunities for real-world evidence in clinical oncology—a view from the UK: proceedings of a national workshop
Source: ESMO Real World Data Digit Oncol. 2024 Nov 18;6:100089. doi: 10.1016/j.esmorw.2024.100089 (PMC12836500; doi:10.1016/j.esmorw.2024.100089)
Supplement: Supplementary data [file mmc1.docx]

# Survey question list

**Role**

Which of the options below best describes your role within Oncology?

Oncologist Physicist Radiographer Dosimetrist Data entry
 Data Management/Digital Health services Other

**Workshop themes:**

1. **Understanding existing attitudes to, and knowledge of, the collection and use of RWD**

How strongly do you agree with the following statements:

“Real-world data can be used to provide high quality evidence to inform clinical decision making.”

Strongly Disagree Disagree Agree Strongly Agree

“The quality of real-world data is too poor for it to be useful.”

Strongly Disagree Disagree Agree Strongly Agree

“Real world data has the potential to provide evidence which clinical trials cannot”
(For example evidence to support treatment of underrepresented patient groups such as the elderly, frail and comorbid, or evidence of the clinical impact of changes in practice too minor to justify a clinical trial)

Strongly Disagree Disagree Agree Strongly Agree

“Changes to the radiotherapy pathway are systematically evaluated in terms of clinical outcomes in my trust.”

All changes Some changes Few changes None

“Staff responsible for data capture understand the value of real world data beyond its primary clinical use”

Strongly Disagree Disagree Agree Strongly Agree Don’t know

“There is an established process for requesting access to real-world data for research purposes at my centre.”

Yes No Don’t know

“Real world data is currently being used for research at my centre”

Yes No Don’t know

1. **Establishing the current status of RWD in terms of data entry processes, content, accessibility, structure and quality control processes**

For the categories listed below please select whether data is stored in a structured or unstructured format. (Structured refers to data stored in named fields as found in a database, whereas unstructured data has no specified format )

Baseline clinical factors:

Structured – all sites Structured – some sites Unstructured Don’t know

Diagnosis & staging:

Structured – all sites Structured – some sites Unstructured Don’t know

Follow-up (outcomes & toxicity):

Structured – all sites Structured – some sites Unstructured Don’t know

In what form(s) are the unstructured data stored? Please select all that apply.

Scan of a physical document Hard copy Text file Image file Other

Are electronic patient reported outcome measures collected at your trust?

Yes – all sites Yes – some sites No Don’t know

Is there a quality control programme in place for real world data in your centre?

Yes No Don’t know

Do you know how to request access to RWD for research purposes in your centre?

Yes No Not sure

Considering both data governance and technical issues, how accessible is RWD following initial clinical use?

Completely inaccessible Difficult to access Accessible Easily accessible Don’t know

If difficult or completely inaccessible, what is the chief cause of this:

Data governance processes Difficulty finding specific records Lack of interoperability between systems Lack of support services Other

1. **Identifying areas of unmet clinical need in which clinical decision making could be supported by RWE**

Please select a site from the options below which reflects your area of specialism or interest (please select none if your role is non-clinical):

H&N Lung Breast Prostate Gastrointestinal Other
None

For the site you have chosen can you identify 1 or 2 clinical scenarios which are poorly supported by current forms of evidence?

[Free text]

Do you think real world data could provide useful evidence for the scenarios identified in the previous question?

Yes No Not sure

# Survey results not featured in the main article

**In what form(s) are the unstructured data stored? Please select all that apply.**

| **Response** | **N** |
| --- | --- |
| Free text in electronic health records | 22 |
| Image file | 8 |
| PDF or other electronic document | 18 |
| Scan of a physical document | 14 |
| Hard copy | 2 |
| Other | 1 |

- Other response: “email”

**Are electronic patient reported outcome measures (ePROMs) routinely collected at your trust?**

**
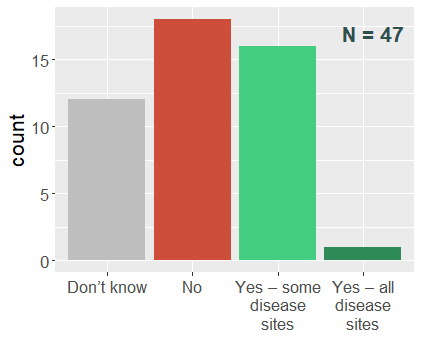
**

**If difficult or completely inaccessible, what are the causes of this:**

**
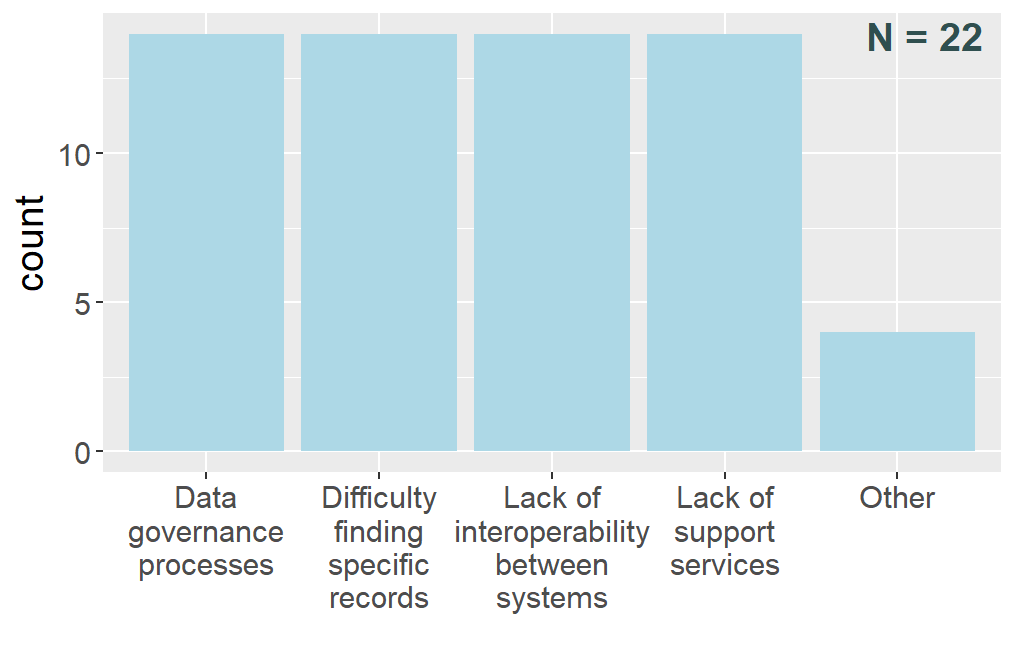
**

- Other responses: "Governance used as an excuse for limiting access. Funding requested for data access (above cost recovery).", "No data export mechanism from EHR or OIS", "N/A", "Lack of statistical support and R&D support"

# Workshop running order

09:30am onwards - Tea, coffee and pastries

10:00am - 12:00pm - **Session 1: Perceptions of the use of Real World Data (RWD)**

- Introduction to the workshop and speakers - Gareth Price (University of Manchester)
- ‘Integration of Real World Evidence (RWE) into NICE processes’ - Stephen Duffield (NICE)
- Pre-workshop survey results - Matt Craddock (University of Manchester)
- Break-out: Current perceptions of using RWD and status of collection and curation of RWD (with tea and coffee)

12:00 - 13:00pm - Lunch

13:00 - 14:30pm - **Session 2: Clinical questions that can be addressed using RWE**

- ‘Real World Evidence and AI for clinical decision support’ - Andre Dekker (Maastricht University)
- Breakout: What are the areas of unmet clinical need where RWE can support clinical decision-making?

14:30 - 15:00pm - Tea and coffee

15:00 - 16:30pm -  **Session 3: Data needed to answer key clinical questions**

- Use of RWD: our infrastructure in Manchester - Corinne Faivre-Finn (University of Manchester)
- What datasets are needed to answer the clinical questions from Session 2 and what are the barriers/solutions to this?

16:30 - 17:00 pm - Sum up (Corinne Faivre-Finn and Gareth Price
